# Supplementary material for: 3D super-resolution optical fluctuation imaging with temporal focusing two-photon excitation
Source: Biomed Opt Express. 2024 Jun 28;15(7):4381–9. doi: 10.1364/BOE.523430 (PMC11249675; doi:10.1364/BOE.523430)
Supplement: Supplementary file 1 [file boe-15-7-4381-s001.pdf]

Supplemental Document

Biomedical Optics EXPRESS

3D super-resolution optical fluctuation imaging with temporal focusing two-photon excitation: supplement

PAWEL SZCZYPKOWSKI,[1,3,] MONIKA PAWLOWSKA,[1,2] AND RADEK LAPKIEWICZ[1,4]

[1] *Institute of Experimental Physics, Faculty of Physics, University of Warsaw, Pasteura 5, Warsaw 02-093, Poland*
[2] *Nencki Institute of Experimental Biology PAS, Pasteura 3, Warsaw 02-093, Poland*
[3] *Pawel.Szczypkowski@fuw.edu.pl*
[4] *Radek.Lapkiewicz@fuw.edu.pl*

This supplement published with Optica Publishing Group on 28 June 2024 by The Authors under the terms of the Creative Commons Attribution 4.0 License in the format provided by the authors and unedited. Further distribution of this work must maintain attribution to the author(s) and the published article's title, journal citation, and DOI.

Supplement DOI: https://doi.org/10.6084/m9.figshare.26001733

Parent Article DOI: https://doi.org/10.1364/BOE.523430

3D Super-resolution Optical Fluctuation Imaging with Temporal Focusing two-photon excitation: supplemental document

– First section of the supplementary material describes analytic derivation of temporal focusing sectioning capability.
– Second section describes staining procedure of cell cultures with quantum dots.
– Third section is the description of the data analysis to get the resolution and the sectioning.

1. TEMPORAL FOCUSING SIMPLIFIED THEORY

We present a simplified theory of Temporal Focusing (TF) to estimate the amount of fluorescence from the out-of-focus planes. The detailed theoretical treatment presented in [1] or [2] is precise but also setup specific. This theory may be useful for quick evaluation of custom design TF setups. The TF setup has three main components: the grating, the lens, and the objective lens. The grating is imaged onto the sample with a 4f imaging telescope. Following the work of Dan Oron et al [2], we align the grating's normal with the optical axis after the grating. This ensures that the temporal focusing plane is not tilted with respect to the imaging plane.

To start our theoretical considerations, we assume that the electric field of the pulse is Gaussian in both spectrum and time. This allows us to derive an analytical expression for the pulse width at any point. To do it, we will neglect all the spatial features of the illuminating beam. Such an assumption is valid for wide-field excitation, as we want to have a large, uniform illumination area. We then transform our problem to 1-D pulse propagation along the optical axis (along z). Such propagation is straightforward in the spectral domain. We just acquire phase proportional to the propagation distance z times the general wave vector $k(\omega)$ along the optical axis - $k_z(\omega)$. In such a model, we can write the complex spectral field depending on the distance as:

$$\tilde{E}(\omega,z) = \tilde{E}_0 exp[-\sigma_t^2(\omega-\omega_0)^2 + ik_z(\omega)z] \tag{S1}$$

Here $\tilde{E}_0$ is a complex spectral amplitude, σ_t is the pulse width in time $\left(E(t) \propto exp(-\frac{t^2}{4\sigma_t^2})\right)$.

As we want to calculate the fluorescence that depends on pulse duration, we need to transform the field from the spectral domain to the temporal domain. To do it, we perform the inverse Fourier transform of expression (S1). For a general function $k_z(\omega)$, this requires numerical methods. Alternatively, we can approximate $k_z(\omega)$ by the Taylor series expansion to the second order:

$$k_z(\omega) \simeq k_0 + \left.\frac{dk_z}{d\omega}\right|_{\omega=\omega_0}(\omega-\omega_0) + \left.\frac{d^2k_z}{d\omega^2}\right|_{\omega=\omega_0}\frac{(\omega-\omega_0)^2}{2} \tag{S2}$$

We can group the terms containing powers of $(\omega-\omega_0)$ and calculate the inverse Fourier transform:

$$E(t,z) = \mathscr{F}^{-1}\left\{\tilde{E}_0 \cdot exp\left[ik_0z + ik_z'z(\omega-\omega_0) - \left(\sigma_t^2 - \frac{ik_z''}{2}z\right)(\omega-\omega_0)^2\right]\right\} = \tag{S3}$$

$$= \frac{\tilde{E}_0}{\sqrt{2(\sigma_t^2 - \frac{ik_z''}{2}z)}} \cdot e^{ik_0z - i\omega_0 t} \cdot exp\left[\frac{(t+k_z'z)^2}{4(\sigma_t^2 - \frac{ik_z''}{2}z)}\right] \tag{S4}$$

We use k_z' and k_z'' to denote the first, and second-order derivatives of k_z at $\omega = \omega_0$. The term e^{ik_0z} is a global phase factor, and we can ignore it. The first-order derivative $ik_z'z$ influences the propagation of the Gaussian envelope. It will also be irrelevant to us. The most important parts are the ones containing k_z''. The pulse width and the peak power are affected by k_z''. We can focus only on influence of k_z'' on the pulse width by writing:

$$E(t,z) \propto \frac{1}{\sqrt{\sigma_t^2 - \frac{ik_z''}{2}z}} \cdot exp\left[-\frac{(t+k_z'z)^2}{4(\sigma_t^2 - \frac{ik_z''}{2}z)}\right]. \tag{S5}$$

Now we can calculate the fluorescence for two-photon absorption. It can be approximated by the integral of the square of the instantaneous excitation intensity [3]:

$$F \propto \int_{-\infty}^{+\infty} I(t)^2 dt, \tag{S6}$$

where we extended the integration limits to infinity for convenience and simplicity. We can use the result from (S5) to get $I(t)$ by taking modulus squared of the field. By inserting the result to S6 we obtain the equation for the fluorescence as a function of the distance from the temporal focus:

$$F \propto \int_{-\infty}^{+\infty} \frac{1}{\sigma_t^4 + \left(\frac{k_z''}{2} z\right)^2} \cdot exp\left[-\frac{(t + k_z' z)^2}{\left(\sigma_t^4 + \left(\frac{k_z''}{2}\right)^2\right)}\right] dt = \frac{\sqrt{\pi}}{\sqrt{\sigma_t^4 + \left(\frac{k_z''}{2} z\right)^2}}. \tag{S7}$$

To estimate the sectioning in the TF setup, we only need to find the second derivative of k_z in our setup. We can approximate $k_z(\omega)$ using geometrical optics. For the central frequency ω_0, the k-vector is parallel to the grating's normal and to the optical axis. The angle of $\vec{k}(\omega)$ after the grating can be expressed as:

$$\alpha(\omega) = 2\pi c N \frac{\omega - \omega_0}{\omega \omega_0}. \tag{S8}$$

Here, c is the speed of light, and N is the grating's groove density. After the grating, light passes through the lens with focal length f_c and then through the objective with effective focal length f_o. The objective and the lens form a 4f telescope, so we have a simple magnification of angles. We can express the tangent of the angle after the objective as:

$$Tan(\gamma(\omega)) = 2\pi c \frac{f_c}{f_o} N \frac{\omega - \omega_0}{\omega \omega_0} \tag{S9}$$

$\frac{f_c}{f_o}$ is the angular magnification of the 4-f telescope (M). We could continue without further approximations, but the calculations would be cumbersome. To simplify them, we can use the paraxial approximation and assume that $Tan(\gamma) = \gamma$. To obtain $k_z(\omega)$, we need to compute the z-axis projection of the k-vector:

$$k_z(\omega) = \frac{n\omega}{c} cos\left(2\pi c M N \frac{\omega - \omega_0}{\omega \omega_0}\right) \tag{S10}$$

We use M instead of $\frac{f_c}{f_o}$ for brevity. In our experiments, we used an immersion objective, and the correct expression for k_z around the temporal focus includes n. n is the refractive index of the medium. Usually, n also depends on the angular frequency ω, but we ignore this dependence due to the short propagation distance through the medium. We then differentiate expression (S10) twice and obtain:

$$\left.\frac{d^2 k_z}{d\omega^2}\right|_{\omega=\omega_0} = \frac{4\pi^2 n c N^2 M^2}{\omega_0^3}. \tag{S11}$$

Simply by plugging the result to (S7) we get the sectioning of TF:

$$F(z) \propto \frac{1}{\sqrt{1 + \left(\frac{2\pi^2 n c N^2 M^2}{\omega_0^3 \sigma_t^4} z\right)^2}} \tag{S12}$$

In this treatment, we assumed that the pulse is the shortest at z = 0. However, we can shift the temporal focus by shifting the position of the grating with respect to the first lens. To estimate the amount of shift, we can use geometric considerations. The sharp image of the object placed at the distance f_1 from the first lens of the 4f system forms at the focus f_o of the objective. When we shift the object by Δx the position of the sharp image shifts by $-(\frac{f_1}{f_2})^2 \Delta x$. The same thing should happen to the temporal focus. We can also shift the temporal focus by adding some group delay dispersion (GDD) to the pulse before the grating. GDD in TF setup is equal to $k_z'' z$. To shift the temporal focus by z_0 μm, we need to add:

$$GDD = \frac{4\pi^2 ncN^2M^2}{\omega_0^3}[fs^2\mu m^{-1}] \cdot z_0[\mu m], \tag{S13}$$

N needs to be in μm^{-1}, $c = 0.2997\mu m \cdot fs^{-1}$, $\omega_0 = 2\pi c/\lambda_0$ $[fs^{-1}]$. Figure S1 shows the good agreement of this simple theory with experiments.

To perform the experiments we used a spin-coated sample of Quantum Dots dissolved in PMMA. We moved the sample along the optical axis, and for every position we took a photo with the camera. We calculated the fluorescence signal as the sum of pixel values over the whole camera sensor. As a result, we got the shape of the fluorescence intensity with respect to the distance from the focus (sectioning). The obtained shape agrees with the theoretical shape from (S12). We used a built-in pulse compressor to change the dispersion of the pulse. For every dispersion value we measured sectioning and fitted $\frac{1}{\sqrt{1+a(z-z_0)^2}}$ (Fig S1a). From the fitted functions we got z_0 - the position of temporal focus. In Fig. S1b the position of the temporal focus with respect to the GDD is presented. We fitted a trend line (blue) which is very similar to the theoretical trend line (orange). In this figure, two points seem to not match the rest of the data. We think it could be caused by the built-in compressor or the reflection of the excitation pulse from the glass slide.

The presented simple theory seems to work even though the pulse is not Gaussian, and not bandwidth-limited. We also used the NA=1.4 objective, where paraxial approximation may not be valid anymore. However, this simple treatment still provides insight into what happens in this system.

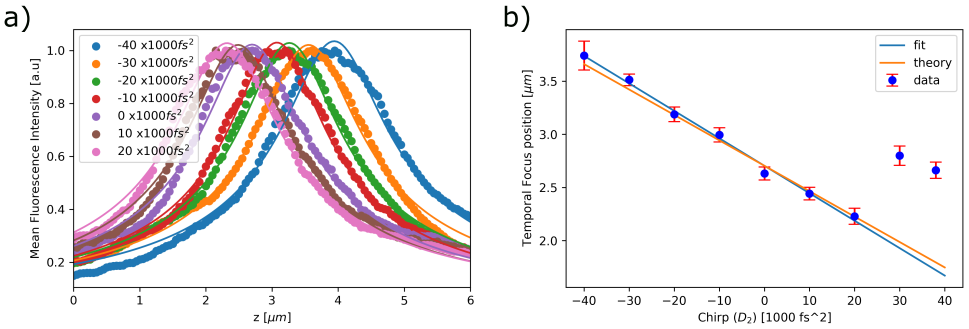

Fig. S1. Temporal focus axial shift with respect to the chirp of an excitation pulse. a) The fluorescence from a thin sample of Quantum Dots spin-coated with PMMA was recorded for different positions with respect to the objective. For every iteration, we introduced a different dispersion value using laser's built-in compressor. We fitted $\frac{1}{\sqrt{1+a(z-z_0)^2}}$ to get z_0 shift that is visible in plot b). We fitted a linear function to the data (blue) to get the offset. The orange line is the theoretical shift with the slope following from the dispersion considerations.

2. STAINING PROCEDURE

SOFI requires fluorescent labels that blink at a rate matched to the camera frame rate. We use quantum dots as the labels because they have high brightness, photostability, and suitable blinking properties. We follow the immunocytochemistry staining protocol adapted from [4], where the authors found that the fluorescence of some quantum dot-conjugated antibodies fades quickly with common buffers. They suggested using a background-reducing agent BKRA (DAKO, S3022) to prevent quenching. The steps of our adapted protocol are as follows:

- We fix three weeks old rat primary dissociated mixed culture with 4% PFA and 4% sucrose.
- We permeabilize the culture with 0.1% triton for 10 minutes.
- We rinse the culture three times with PBS.
- We incubate the culture in 10% normal donkey serum (Sigma-Aldrich®, S30) for blocking (1.5h).

- We stain the culture with Microtubule Associated Protein 2 (MAP2) antibody diluted in PBS with 2% normal donkey serum overnight at 4 deg C.
- We wash the culture in BKRA solution 1/50 in 50mM Tris, as recommended in [4].
- We incubate the culture with Donkey anti-Mouse Secondary Antibody, Qdot625 (Invitrogen™ Q22085) diluted 1:300 in BKRA for 1h at room temperature.
- We rinse the culture three times in BKRA solution (1/50 in 50mM Tris).
- We mount the coverslip with ProLong™ Glass Antifade Mountant with NucBlue™ (Invitrogen™, P36981).

This staining procedure allowed us to perform experiments for several days after the staining. In the first days, we observed the blinking Qdots that had a granular structure. After about two weeks, the granular structure disappeared, the fluorescence weakened and hardly any SOFI signal could be detected.

3. RESOLUTION MEASUREMENTS

A. Lateral Resolution

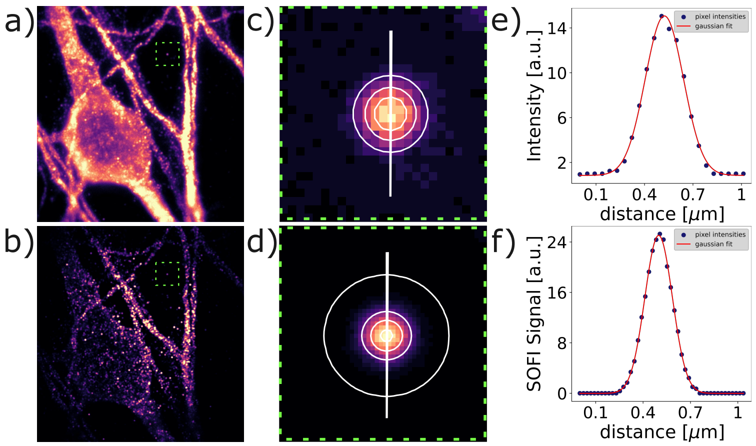

Fig. S2. (a),(b) – mean intensity image and SOFI image respectively. (c), (d) – magnified areas around point emitter. (e), (f) – cross-section along the vertical white line with fitted Gaussian function (red). On the images a) and b) the green rectangles have size of around $3\mu m \times 3\mu m$. The size of images (c), (d) is around $1.15\mu m \times 1.2\mu m$.

To measure the resolution we used the mean intensity images and SOFI images presented in the work. We searched for regions that had single emitters. We then took a cross-section through the center of the emitter and fitted a Gaussian function. We measured 10 such points from which we estimated the resolution of SOFI to be 199 ± 12 while the resolution of the mean intensity image is 274 ± 18 nm. The obtained value for the mean intensity image is similar to the theoretical one. This ensures that we take single emitters or the ones that cannot be resolved. The measured values are placed in table S1. The images of single emitters are also available in [5]. In figure S2 the exemplary point emitter is chosen from the fields of view (a) and (b) corresponding to the mean intensity image and SOFI image respectively. We fit 2D Gaussian to point emitters from the mean intensity image and SOFI image – (c) and (d). The vertical cross-section through the point emitter can be approximated by the Gaussian function. The results from 2D fit and 1D fit are consistent within 5% error.

	mean image d	sofi image d	FWHM mean	FWHM SOFI
	119,6	86,8	281,6	204,3
	114,8	83,4	270,4	196,3
	108,7	82,9	256,0	195,1
	125,0	88,6	294,4	208,7
	122,4	90,9	288,3	214,1
	112,8	79,8	265,7	187,8
	103,8	77,6	244,4	182,8
	128,4	94,3	302,4	222,1
	121,8	86,9	286,7	204,6
	115,2	82,7	271,3	194,8
	116,9	79,1	275,3	186,2
	108,2	80,9	254,9	190,6
Mean	116,5	84,5	274,3	198,9
Standard deviation	7,4	5,1	17,3	12,0

Table S1. Table with values of Gaussian widths and FWHM's to single emitters. Value "d" corresponds to the Gaussian width with following definition: $A \cdot exp(\frac{(x-x_0)^2}{2d^2}) + C$.

B. Sectioning Measurements

To measure the sectioning of SOFI we chose different regions of the SOFI z-scan to decrease the potential influence of the slight tilt of the sample. To every z-scan data of the chosen regions of interest (normalized average of the SOFI signal for every z-position), we fitted the Gaussian function in the form of $\cdot exp(\frac{(x-x_0)^2}{2d^2})$. We discarded two fits from 10, as they had larger values on the diagonal of the covariance matrix than other measurements. We calculated the mean of the fitted Gaussian widths "d" and the corresponding standard deviation. This resulted in the average of SOFI FWHM being 481 ± 14 nm. The Gaussian function of the same FWHM is plotted in Figure 3 in the manuscript. The amplitude of this Gaussian is not equal to 1 to better fit the data points. The data points for the SOFI signal correspond to the average of the normalized data from different regions of the image. Because the sample was probably tilted or uneven, the maximum signal wasn't the same for 0 axial position (defocus) for different regions of interest. This results in the average of the normalized SOFI signal not reaching 1. This also causes the potentially fitted Gaussian to this data to be slightly wider (500 nm FWHM) than the one we calculated with the averaging of d's obtained for different regions of the image.

For TF sectioning we gathered the data from regions of the sample where the quantum dots weren't clustered. Similar, to fitting with SOFI, we fitted the function $F = \frac{1}{\sqrt{1+a(z-z_0)^2}}$ for every z-stack data, and calculated the mean and the standard deviation of the resulting FWHM. In figure 3 in the manuscript We plotted the theoretical curve with expected FWHM. For the TF data points we took the average of the normalized data for different regions of interest – applying the same procedure as we did for the SOFI signal.

REFERENCES

1. G. Zhu, J. van Howe, M. Durst, *et al.*, "Simultaneous spatial and temporal focusing of femtosecond pulses," Opt. Express **13**, 2153–2159 (2005).
2. D. Oron, E. Tal, and Y. Silberberg, "Scanningless depth-resolved microscopy," Opt. Express **13**, 1468–1476 (2005).
3. M. Göppert-Mayer, "Über elementarakte mit zwei quantensprüngen," Annalen der Physik **401**, 273–294 (1931).

4. S. Prost, R. E. B. Kishen, D. C. Kluth, and C. O. C. Bellamy, “Working with Commercially Available Quantum Dots for Immunofluorescence on Tissue Sections,” PLoS ONE **11** (2016).
5. P. Szczypkowski, “Data supporting 3D Super-resolution Optical Fluctuation Imaging with Temporal Focusing with two-photon excitation,” https://doi.org/10.5281/zenodo.11094685. (2024).
